# *Moringa Oleifera* Leaf Extract Repairs the Oxidative Misbalance following Sub-Chronic Exposure to Sodium Fluoride in Nile Tilapia *Oreochromis niloticus*

**DOI:** 10.3390/ani10040626

**Published:** 2020-04-05

**Authors:** Nirmen F. Ahmed, Kadry M. Sadek, Magdy Kh. Soliman, Reyad H. Khalil, Asmaa F. Khafaga, Jamaan S. Ajarem, Saleh N. Maodaa, Ahmed A. Allam

**Affiliations:** 1 Department of Biochemistry, Faculty of Veterinary Medicine, Damanhour University, Damanhour 22511, Egypt; Nirmenfathi@gmail.com (N.F.A.); ksaadek@gmail.com (K.M.S.); 2 Department of Poultry and Fish diseases, Faculty of Veterinary Medicine, Damanhour University, Damanhour 22511, Egypt; m.khalil@gmail.com; 3 Department of Poultry and Fish diseases, Faculty of Veterinary Medicine, Alexandria University, Edfina 22758, Egypt; Riad.Khalil@alexu.edu.eg; 4 Department of Pathology, Faculty of Veterinary Medicine, Alexandria University, Edfina 22758, Egypt; 5 Department of Zoology, College of Science, King Saud University, Riyadh 11451, Saudi Arabia; jajarem@ksu.edu.sa (J.S.A.); maodaa_28@yahoo.com (S.N.M.); 6 Department of Zoology, Faculty of Science, Beni-suef University, Beni-suef 65211, Egypt; Allam1081981@yahoo.com

**Keywords:** *Moringa* ethanolic extract, NaF, Nile tilapia, malondialdehyde, antioxidants, GST mRNA expressions

## Abstract

**Simple Summary:**

The present study investigated the antioxidant effect of ethanolic extract of *Moringa Oleifera* (MO) leaves against sodium fluoride-induced toxicity in *Nile tilapia*. It is concluded that MO leaves are a promising antioxidant plant via downregulation of lipid peroxidation, and upregulation of antioxidant enzyme activity including SOD, CAT, GPx, GSH, and TAC in liver, kidney, gills, and muscle tissue in a time-dependent manner, in addition to downregulation of mRNA expression of antioxidant-related genes.

**Abstract:**

The potential antioxidant property of *Moringa oleifera* (MO) has been the recent focus of an increased number of studies. However few studies investigated its antioxidative ability against sodium fluoride-induced redox balance breakdown in *Oreochromis niloticus*. Thus, this study evaluates the effects of MO against the oxidative stress induced by sub-chronic exposure to sodium fluoride (NaF). A total of 264 fish (40 ± 3 g BW) were used to calculate the 96 hr-LC50 of NaF and perform the sub-chronic exposure study. 96 hr-LC50 of NaF was calculated as (61 mg/L). The 1/10 dose of the calculated 96 hr-LC50 (6.1 mg/L) was used to complete the sub chronic exposure for eight weeks. Fish were divided into four groups (n = 51; three replicates each); control, non-treated group; NaF group (exposed to NaF 6.1 mg/L); MO group (treated with 1% MO of diet); and NaF+MO (exposed to NaF 6.1 mg/L and treated with 1% MO of diet). The results revealed that the sub-chronic exposure to NaF (6.1 mg/L) was substantially increased malondialdehyde (MDA) and decrease the activities of superoxide dismutase (SOD), catalase (CAT), glutathione reduced (GSH), glutathione peroxidase (GPx), and total antioxidant capacity (TAC) in the gills, liver, kidney, and muscle tissue in a time-dependent manner. In addition, a significant reduction in mRNA expression of GST in the liver was reported following NaF exposure. On the contrary, dietary supplementation of MO to NaF-exposed fish resulted in a significant reduction in MDA levels, and a significant elevation of SOD, CAT, GSH, GPx, and TAC activities in a time-dependent manner, in addition to significant elevation of GST mRNA expression in liver tissue. It could be concluded that a 1% MO (*w*/*w*) ration is a promising antioxidant plant that may successfully use to interfere with the oxidation processes induced by NaF in various tissues of *Oreochromis niloticus*.

## 1. Introduction

Fluoride is a nonmetallic negatively-charged ion. It occurs naturally in the rocks, soil, and water of seas, rivers, and lakes [1]. The concentrations of fluoride in polluted seawaters generally range from 1.2 to 1.5 mg F/L [2]. The dramatic increases in industrial waste, municipal sewage, and the use of sodium fluoride-based insecticides can result in an increase of fluoride concentration in aquatic media which, in turn, leads to fluoride toxicity and accumulation in fish tissues [3,4]. These increases in fluoride toxicity in fish are positively correlated with the increasing concentration of aquatic medium fluoride, water temperature, and exposure time [4]. Interestingly, elevated concentrations of fluoride were reported to delay the hatching of fertilized eggs of freshwater fish [5], inhibit fish growth, such as length and weight [6], and accumulated in fish bone, gill, cartilage, and skin [7]. These findings may suggest the economic and medicinal needs to find suitable herbal remedy to reduce these adverse effects of fluoride on fish. The exposure to high concentrations of fluoride could trigger the adverse toxic impact on different biological processes [8]. In different words, low concentration of fluoride (*μ*M) might act as a promoter for cell proliferation and enzymatic activity; however, a high concentration of fluoride (mM) could act as an enzymatic inhibitor. This enzyme inhibitory action occurred due to the strong electronegativity of fluoride, where it forms ions and interacts with enzymes leading to toxicity and biological damage to different body systems [9], such as the central nervous system [10], spleen and immune organs [11], the male reproductive system [12], and the liver [13,14]. 

Few previous studies concluded the role of fluoride as a toxic, cumulative, environmental pollutant in aquatic life [8] where they are regularly exposed to elevated concentrations of fluoride in surface waters. The adverse impact of fluoride was ensured when it enters the food chain and accumulates in different tissues of aquatic organisms, particularly fish bone and the invertebrate’s exoskeleton. In addition, it can disturb the normal metabolic homeostasis and exert its adverse effect even at very low levels. Recent evidence in fish models indicated that high concentrations of fluoride can modify growth and development, induced pathologic bone deformity, and disrupt cell respiration and metabolism [15,16,17]. These events lead to an excessive production of reactive oxygen species (ROS) with resultant impairment of antioxidant system, DNA damage, mitochondrial dysfunction, and increased mortality rate [18,19].

*Moringa oleifera* (MO) is an edible tree, belonging to the Moringaceae family. It is present in tropical and subtropical areas of Asia and Africa. MO is known for its nutritional and pharmacological importance [20,21,22]. All over the world, MO has been extensively and traditionally used to alleviate several afflictions, such as hepatorenal, cardiovascular, hematological, and gastrointestinal disorders. In addition, it has efficient anti-inflammatory, antimicrobial, and anti-oxidative properties [22,23,24]. Natural herbalists use the different parts of MO, such as leaves, roots, flowers, fruit, and seeds, to obtain different extracts of *Moringa*. Among them, *Moringa* leaf extract is known for its rich contents of calcium, potassium, iron, phosphorous, and vitamins (vitamins A, D, C, E, ascorbic acid, and β-carotene), in addition to natural antioxidants and phytochemical flavonoids, such as polyphenol oxidase, oxidase, and catalase, which has great medicinal importance [20,25,26,27]. Recent studies focused on the use of MO leaf meal in fish feeds as a cheap and locally available plant, with increasing protein contents [28].

Nile tilapia, *Oreochromis niloticus*, is a type of freshwater fish that is commonly used in toxicological investigations [29]. It is characterized by resistance to diseases, fast growth, and easy adaption to commercial diets [30]. Several studies were performed to evaluate the importance of medicinal plants’ leaf extracts for detoxification of polluted ecosystems [31,32,33,34,35], promote fish growth [36], and improve resistance against toxicity. However, in one recent study which has evaluated the ameliorative effect of MO leaf extract against sodium fluoride (NaF) in sea bream via the estimation of liver function, catalase, and superoxide dismutase activity [37], the authors concluded an efficient hepatoprotective and antioxidant effect of MO leaf extract. However, more investigation is needed to explain the anti-oxidative efficacy of MO ethanolic extract against NaF toxicity in aquatic organisms. Hence, the present investigation was conducted to assess the antioxidant and antigenotoxic ability of MO against NaF-induced oxidative stress via evaluation of oxidative stress biomarkers (malondialdehyde (MDA), superoxide dismutase (SOD), catalase (CAT), glutathione peroxidase (GPx), glutathione reduced (GSH), and total antioxidant capacity (TAC)) in various tissues (liver, kidney, gills, and muscle), and via the molecular evaluation of the antioxidant related gene (glutathione S transferase (GST) in the liver of Nile tilapia as a fish model.

## 2. Materials and Methods

All procedures by this study were in accordance with international ethical standards. The research involved no human participants.

### 2.1. Chemicals

Sodium fluoride (NaF; ≥99%; MW 41.99; EC No. 231-667-8) was purchased from Sigma-Aldrich (Hamburg, Germany). Kits for malondialdehyde (Cat# MD 2529), Superoxide dismutase (Cat# SD 2521), catalase (Cat# CA2517), glutathione peroxidase (Cat# GP 2524), glutathione reduced (Cat# GR 2511), and total antioxidant capacity (Cat# TA 25 13) were obtained from Biodiagnostic Co., Cairo, Egypt. All chemicals were of the highest available analytical grades.

### 2.2. Preparation of MO Leaf Ethanolic Extract 

The fresh leaves of the MO plant were obtained by personal communication from El-Sharkia Governorate, Egypt. The collected leaves were purified, washed with distilled water, and dried in a shed for two weeks, until they become crispy in touch while retaining their greenish coloration. Later, ethanolic extract of MO leaves was prepared according to the previously described method [38]. Briefly, 1000 g were macerated in absolute ethyl alcohol and distilled water (ethanol 80 %) and left for 48 h. The resulting extract was filtered through muslin cloth on a plug of glass wool in a glass column, concentrated, and evaporated via a rotary evaporator at 45 °C to dryness to prevent the active ingredients’ denaturation. Following that, the extract was diluted to 1000 mL (polysaccharide was used as a carrier) and stored in a refrigerator.

### 2.3. Gas Chromatography–Mass Spectrometry(GC-MS) Analysis

**Figure 1:**
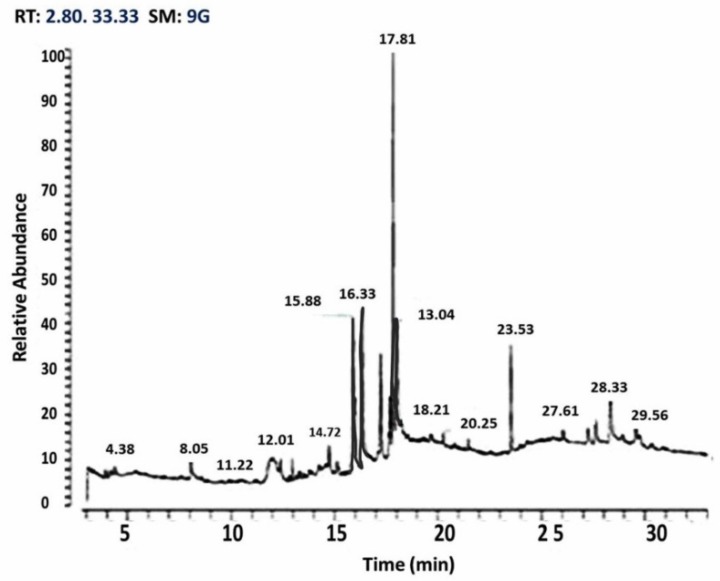
The mass spectra of the identified components of MO as compared to NIST 11 mass spectral database.

A trace GC Ultra-ISQ mass spectrometer with a direct capillary column TG–5MS (30 m × 0.25 mm × 0.25 μm) was injected by 10 μL of MO ethanolic extract. The column oven temperature was started at 60 °C and then increased by 5 °C/min until reaching 280 °C. The injector and detector (MS transfer line) temperatures were kept at 250 °C. A helium flow rate of 1 mL/min. was used as the carrier gas for 37.83 min. The solvent delay was 2 min. and diluted samples of 1 μL were injected automatically using auto-sampler AS3000 coupled with GC in the splitless mode. The ion source and quadrupole temperatures were set at 200 and 150 °C, respectively. The mass spectra of the identified components were determined by comparison to NIST 11 mass spectral database (Figure 1).

### 2.4. Fish Maintenance

A total of 264 apparently healthy and uniform size and weight *O. niloticus* (with average body weight 40 ± 3 g) were obtained from the Central Laboratory of Abbassia Fish Farm, Egypt. Fish were acclimated for 14 days in full glass aquaria measuring (40 × 30 × 40 cm) and maintained under standard laboratory condition (oxygen saturation 90–95%, pH 6.5, water temperature of 25 ± 2 °C, total hardness 150 mg/L, and 12:12 dark/light cycle). Fish were fed on a commercial fish pellet diet containing 25% crude protein. The diet was provided daily at a fixed feeding ratio of 3% of the body weight of the fish [39].

### 2.5. Determination of 96-h LC50 of Sodium Fluoride 

After acclimation period, a total number of 60 apparently health fish were selected and allocated into six equal groups (n = 10 in two replicates; each replicate contains five fish). Fish were consistently exposed to 0, 20, 40, 60, 80, and 100 mg/L of NaF. The behavioral, clinical, and post-mortem changes were closely followed up and recorded during the experiment. Mortalities were checked every 24 h and the dead fish were immediately removed. The 96-h LC50 of sodium fluoride was determined and calculating according to Behrens and Karber [40]. 

### 2.6. Experimental Design 

Fish were randomly divided into four equal groups (51 fish per each). Each group consisted of three replicates (17 each). The 1st group served as control non-treated group. The 2nd group supplemented with MO leaf extract (1% (*w*/*w*) ration). The 3rd group exposed to NaF (1/10 LC50; 6.1 mg/L) according to Singh et al. [41]. The 4th group exposed to NaF (6.1 mg /L) and supplemented with MO (1%) in ration (*w*/*w*). The experimental period was extended up to eight weeks, where samples were collected every two weeks from all aquaria for analyses. During the experimental period, the water of the aquaria changed daily with clean aerated water contains the same concentrations of NaF. Fish were kept in static fiberglass tanks with side channel blowers to blow the air that was diffused into each tank with an air stone. The tested pellet diet was prepared by mixing the commercial fish diet with 1% (*w*/*w*) MO leaf extract powder and a little water, then the mixture was run through a pasta maker and the pellets placed onto a cookie sheet. Following that, pellets were dried on low heat in an oven. The diet was administrated to fish twice daily at 08:00 and 13:00.

### 2.7. Sample Collection and Preparation

At the 2nd, 4th, 6th, and 8th weeks of the experiment, twelve fish were collected from each treated group (4 fish/replicate), fish were anaesthetized with 0.02% benzocaine solution and tissue samples were collected from the gills, liver, kidney, and muscle and stored at −20 °C for further evaluation of oxidant/antioxidant parameters. Stored tissue samples (gills, liver, kidney, and muscle) were washed in an ice cold 1.15% KCl solution, blotted, weighed, and homogenized with 0.1 M phosphate buffer (pH 7.2). Later, tissues were put in a mortar and blended with acid-washed laboratory sand with a pestle. The resulting homogenate was centrifuged at 2500 rpm for 15 min. The supernatant was decanted and stored at −20 °C.

### 2.8. Estimation of Antioxidative/Antioxidative Parameters in Tissues Homogenate 

The stored samples of tissue homogenate were used for spectrophotometric determination of antioxidant enzymes activity using a UV–VIS spectrophotometer. Malondialdehyde (MDA) levels were estimated in tissues homogenate samples using commercially available colorimetric kits. The assay relied on the reaction between MDA and thiobarbituric acid at absorbance of 532 nm to form a pink-colored complex [42]. Superoxide dismutase (SOD, EC 1.15.1.1) activity was colorimetrically evaluated using laboratory-supplied kits; the assay based on the inhibition of nitroblue tetrazolium dye reduction through phenazine methosulphate enzyme [43]. CAT activity (CAT, EC 1.11.1.6) was estimated using commercially supplied kits by interaction with H_2_O_2_, and then inhibition of this reaction by CAT inhibitor [44]. In addition, activity of glutathione peroxidase (GPx, EC 1.11.1.9) was estimated according to Paglia and Valentine [45] using commercially available kits. Furthermore, levels of glutathione reduced (GSH) was colorimetrically evaluated using laboratory provided kits, the principle of assay based on the measuring the conjugation of 1-chloro-2,4-dinitrobenzene with reduced glutathione at an absorbance of 340 nm [46]. The total antioxidant capacity (TAC) was spectrophotometrically estimated in tissue homogenate samples using laboratory-supplied kits according to the manufacturer’s instructions.

### 2.9. Evaluations of RT-PCR mRNA Expressions of Glutathione S Transferase Genes

At the end of the experiment, liver samples were collected from different groups to perform the real-time reverse transcription polymerase chain reaction (RT-PCR) analysis of mRNA expression of the antioxidant related gene (glutathione S transferase; GST). The total mRNA was extracted from six liver samples/group using mRNA extraction kit (QIAamp^®^ RNeasy Mini kit; QIAGEN GmbH, Hilden, Germany) according to the manufacturer’s instructions. One hundred milligrams of each live sample was added to 600 μL of RLT buffer (containing 10 μL β-mercaptoethanol/mL). Samples were homogenized by placing the tube into the adaptor sets fixed into the clamps of a Qiagen tissue lyser. Digestion of DNase was applied for 15 min at 30 °C to obtain highly pure RNA and remove any DNA residue. The quality of the extracted RNA was confirmed using 2% agarose electrophoresis according to the manufacturer’s instruction. Real-time RT-PCR was performed via use the QuantiTect SYBR Green PCR Master Mix (Qiagen, Germany, GmbH). A 25-μL reaction of GST gene was prepared by 12.5 μL of 2× QuantiTect SYBR Green PCR Master Mix (QIAGEN GmbH, Hilden, Germany), 0.25 μL of RevertAid Reverse Transcriptase (200 U/μL) (Thermo Fisher scientific, Milano, Italy), 0.5 μL of primer at an amount of 20 pmol, 8.25 μL of water, and 3 μL of RNA template. The primer sequences for the target genes are described in Table 1. Stratagene MX3005P real-time PCR machine was used to perform the reaction. Amplification curves and threshold cycle (Ct) values were determined using Stratagene MX3005P software. The Ct value of each sample was compared with the positive control samples to estimate variations in mRNA expression among the different samples according to the 2-∆∆Ct method described by Yuan et al. [47].

### 2.10. Statistical Analysis 

The collected data were analyzed statistically with general linear model (GLM) using 4 × 4 factorial design according to the following model: Xifk = μ + Ti + Pf + Ti × Pf + Eifk, where: Xifk = the value of any observation, μ = the population mean Ti = treatment effect (i = control (CTR) – sodium fluoride (NaF) – *Moringa oleifera* (MO) – NaF + MO), Pf = Time effect (f = 2, 4, 6, and 8 weeks), Ti × Pf = the interaction between the treatment and the time effect, and Eifk = random error. Levene’s test was firstly conducted to assess the normality of data. Differences among means considered significant at level (*p* < 0.05). Data were also tested for normality using the Kolmogorov–Smirnov test and considered normally distributed at *p* > 0.05 (*p* = 0.11). SPSS statistical package version 17.0 for Windows (IBM, Armonk, NY, USA) was used for all data analysis. 

## 3. Results

### 3.1. The Chemical Composition of MO

As illustrated in Table 2 and Figure 1, GC–MS analysis of MO revealed that the major phytoconstituents present in Moringa oleifera leaves ethanolic extract are: eugenol (45.23%), caryophyllene (11.35%), hexadecanoic acid (7.23%), phenol (6.14%), octadecenoc acid (3.29%), heptadecyne (3.18%), cyclopropanoctonic (4.17%), heptatriacotanole (1.26%), and quercetin (0.89%).

### 3.2. Determination of LC50 Sodium Fluoride (NaF) in *O. niloticus*

**Figure 2:**
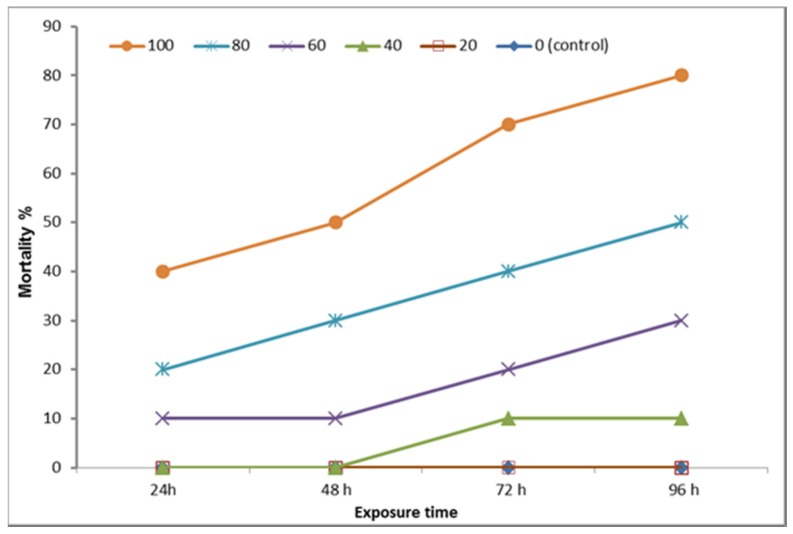
Mortality percentage of fish exposed to different concentration of NaF (20, 40, 60, 80, and 100 mg/L) at different time points (24, 48, 72, and 96 h). The total number of fish/group = 10 fish.

As shown in Figure 2 and Table 3, the lethal concentration 50 (LC50) of NaF in *O. niloticus* has been calculated as 61 mg/L; therefore, the 1/10 dose of NaF LC50 was estimated as 6.1 mg/L.

### 3.3. Effect of NaF and/or MO on Malondialdehyde Levels (MDA) in Tissues Homogenate

As shown in Table 4, the levels of MDA in gills tissues showed significant elevation in NaF and NaF+MO exposed fish from the 4th until the 8th week of the experiment compared to their control counterparts. However, MO treated fish showed significant reduction in MDA levels from the 4th until the 8th week of experiment compared to the other groups. This significant up-regulation was time-dependent where it increased significantly with the increment of exposure period. The same trend of MDA level in gills tissues were reported for the liver, kidney, and muscles. MDA significantly increased in NaF and NaF + MO groups but significantly decreased in the MO group compared to the control fish, with a time-dependent significant increment. 

### 3.4. Effect of Sodium Fluoride and/or *M. oleifera* on Glutathione Reduced (GSH) Activity in Tissue Homogenate 

As shown in Table 5, the levels of GSH in gills tissues showed significant reduction in NaF and NaF + MO groups but GSH significantly increased from the 6th until 8th weeks of experiment, respectively, compared to the control group. This significant downregulations was time-dependent, where it decreased significantly with the increment of exposure period.

Levels of GSH in liver tissues showed significant downregulation in NaF and NaF + MO group from the 2nd weeks of experiment and along the period of experiment compared to their control counterparts. However, fish of MO treated group showed significant elevation in GSH levels at the 4th week of the experiment compared to control and other treated groups. This significant downregulation was time-dependent, where it decreased significantly with the increment of exposure period. Concerning the levels of GSH in kidney and muscles tissues, significant reduction was reported in NaF and NaF + MO group from the 4th until the 8th week of experiment, respectively, and along the period of experiment compared to their control counterparts. However, fish treated with MO showed significant elevation in GSH levels for the same period of experiment compared to the control and other treated groups. This significant downregulation was time-dependent, where it decreased significantly with the increment of exposure period.

### 3.5. Effect of NaF and/or MO on Glutathione Peroxidase Activities (GPx) in Tissues Homogenate

As illustrated in Table 6, the levels of GPx in gills tissues revealed non- significant difference in NaF and NaF + MO groups along the experimental period compared to control counterparts. Moreover, fish treated with MO showed a non-significant elevation in GPx levels along the experimental period compared to control group. Concerning the exposure period, a significant difference was detected at the 8th week only compared to the 2nd week. Levels of GPx in liver and muscle tissues showed significant downregulation in NaF and NaF + MO groups from the 2nd until the 8th weeks of experiment compared to their control counterparts. However, fish treated with MO showed non-significant elevation in GPx levels along the experimental period compared to their counterparts in NaF group. This significant downregulation was time-dependent, where it decreased significantly with the increment of exposure period. Concerning the levels of GPx in kidney tissues, MO showed significant elevation in GPx levels from the 4th until the 8th weeks of the experiment compared to other group. Concerning the exposure period, significant difference was detected at the 8th week only in NaF-exposed fish compared to their counterparts at the 2nd week.

Levels of GPx in muscle tissues showed significant decreased in NaF and NaF + MO groups from the 6th and 8th weeks of experiment, respectively, and along the period of experiment compared to their control counterparts. However, fish treated with MO showed significant increase in GPx levels at the 8th week of the experiment compared to NaF and NaF + MO groups. Concerning the exposure period, non-significant difference was detected for different groups along the experimental period.

### 3.6. Effect of Sodium Fluoride and/or *M. oleifera* on Catalase (CAT) Activity in Tissue Homogenate of *O. niloticus* Gills, Liver, Kidney, and, Muscle

As illustrated in Table 7, the levels of CAT in gills tissues was significantly increase in MO from the 2nd until the 8th week of experiment compared to other treated and control counterparts. Concerning the exposure period, significant difference was detected at the 6th and 8th weeks in NaF and NaF group compared to the 2nd week. Levels of CAT in liver tissues showed significant downregulation in NaF and NaF + MO groups from the 4th week of experiment and along the period of experiment compared to their control counterparts. However, fish of MO group showed significant elevation in CAT levels compared to their counterparts in NaF and NaF + MO groups from 4th until the 8th weeks of the experiment. There is significant downregulation was time-dependent, where it decreased significantly with the increment of exposure period. Concerning the levels of CAT in kidney and muscles tissues, significant reduction was reported in NaF and NaF + MO and MO groups at the 8th week of experiment compared to their control counterparts. Concerning the exposure period, no significant difference was detected in all groups of fish along the experimental period. 

### 3.7. Effect of Sodium Fluoride and/or *M. oleifera* on Super Oxide Dismutase (SOD) Activity in Tissue Homogenate 

As illustrated in Table 8, the levels of SOD in gills tissues showed significant decrease in NaF and NaF + MO group fish at the 4th week of experiment and along the experimental period compared to control counterparts. Moreover, fish of the NaF + MO group showed a non-significant elevation in SOD levels along the experimental period compared to their counterparts in the NaF exposed group. Concerning the exposure period, significant difference was detected at the 6th and 8th weeks of experiment in the NaF and NaF + MO groups, respectively, compared to the 2nd week. Moreover, levels of SOD in the liver, kidneys, and muscle tissues showed significant downregulation in NaF and NaF + MO groups of fish from the 2nd and 4th weeks of experiment, and along the period of experiment compared to their control counterparts. However, fish of the NaF + MO group showed a non-significant elevation in SOD levels along the experimental period compared to their counterparts in the NaF-exposed group. This significant downregulation was time-dependent, where it decreased significantly with the increment of exposure period.

### 3.8. Effect of Sodium Fluoride and/or *M. oleifera* on Total Antioxidant Capacity (TAC) Activity in Tissue Homogenate 

As illustrated in Table 9, the levels of TAC in gills and liver tissues was significantly reduced in NaF and NaF + MO groups at the 6th week of experiment and along the experimental period compared to control counterparts. Moreover, fish co-treated with MO showed significant elevation in TAC levels at the 8th week of experiment compared to their counterparts in the NaF-exposed group. Concerning the exposure period, a significant difference was detected at the 4th, 6th, and 8th weeks of experiment in NaF-exposed fish compared to the 2nd week. 

Concerning the levels of TAC in kidney tissues, significant reduction was reported in NaF and NaF + MO group fish at the 8th experiment compared to their control counterparts. Additionally, fish of the NaF + MO group showed a significant elevation in TAC levels at the 8th week of experiment compared to their counterparts in the NaF-exposed group. Concerning the exposure period, significant difference was detected at the 4th and 6th week of experiment in the NaF-exposed fish compared to their counterparts at the 2nd week. Levels of TAC in muscle tissues showed a significant decrease in the NaF and NaF + MO groups at the 6th and 8th weeks of experiment, compared to their control counterparts. However, fish NaF + MO group showed significant reduction in TAC at the 6th and 8th weeks of the experiment compared to their counterparts in NaF-exposed group. Concerning the exposure period, a significant difference was detected in NaF-exposed fish at the 6th and 8th week of experiment compared to their counterparts at the 2nd week. Fish receiving MO alone showed a significant upregulation in tissue TAC levels throughout the experiment in a time-dependent manner.

### 3.9. Quantitative Analysis of mRNA Abundance of Glutathione S Transferase (GST) Genes in Liver Tissues of *O. niloticus*

**Figure 3:**
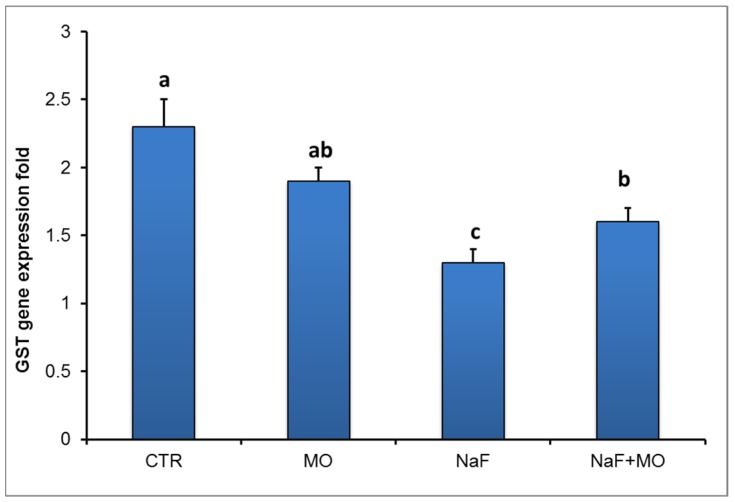
Effect of *Moringa oleifera* (MO) leaves extract on glutathione S transferase mRNA expression in *O. niloticus*. Six samples were analyzed to obtain an average concentration for each treatment.

As illustrated in Figure 3, the exposure of *O. niloticus* to NaF for eight weeks resulted in significant downregulation (*p* < 0.001) of GST gene expression, whereas the corresponding group of fish treated with *M. oleifera* were indistinguishable from controls. The administration of MO in combination with NaF resulted in a significant upregulation (*p* < 0.001) of GST gene expression compared to fish administrated NaF alone.

## 4. Discussion 

Fluoride occurs normally in unpolluted waters at low concentrations (approximately 0.01–0.3 mg/L). However, this level could increase more than 100 times due to the anthrogenic activities of humans [48]. The increased fluoride concentration usually leads to adverse impact on the cells of aquatic organisms due to various enzyme inhibitions [9]. These enzymatic responses are related to the increased production of reactive oxygen species (ROS) leading to oxidative stress [49,50]. In fish, oxidative stress can be defined as an imbalance between oxidants and antioxidant defences [51]. Excessive production of ROS is associated with severe reactions, such as damage of cellular proteins, lipids, and nucleic acid [52]. In addition, ROS can initiate the lipoperoxidation, which is a self-propagating process that leads to formation of peroxyl radical due to the reaction of ROS with the hydrogen atom from an intact lipid [52]. 

Although fish are able to accumulate fluoride through the food chain, few reports have investigated the adverse impacts of fluoride in fish [53]. For example, fluoride is able to delay the hatching of fertilized eggs, inhibit growth, and accumulates in the bone, gill, cartilage, and skin of fish [5,48] and, importantly, it can promote oxidative damage by directly increasing the cellular concentration of ROS by altering the cellular antioxidant capacity in fish [54]. In the current study, the lethal concentration 50 (LC50) of sodium fluoride in *O. niloticus* was calculated as 61 mg/L; therefore, a 1/10 dose of LC50 (6.1 mg/L) was used to induce sub-chronic toxicity. A simple comparison with the previously calculated LC50 values in different species revealed that a 96 hr LC50 was as low as 10.5 mg/L for the saltwater mysidacean *Mysidopsis bahia* [55]. However, the rainbow trout *Oncorhynchus mykiss* seems to be more sensitive to fluoride toxicity than other freshwater fish species. This difference in LC50 values may be attributed to the statement that the aquatic organisms living in soft waters may be more adversely affected by fluoride pollution than those living in hard or seawaters, where increasing water hardness leads to a reduction of fluoride bioavailability of fluoride ions due to the formation of innocuous complexes such as CaF2, Ca5 (PO4)3F, and MgF2 [56].

The findings of the sub-chronic toxicity in this study indicate the adverse oxidative effect of sodium fluoride in the gills, liver, kidney, and muscle tissues. Fluoride has a small ionic radius and high biological activity; therefore, it can penetrate easily into the cells and exerting its adverse effects on various tissues [57]. In addition, some metals, including fluoride, are known for its powerful oxidant effect; they can deplete the major antioxidants in the cell, particularly thiol-containing antioxidants enzymes [49]. Interestingly, the sodium fluoride-related oxidative stress was defined in a time-dependent manner. This time-related progression in the described oxidative stress may be attributed to the exhaustion of the antioxidant system via continuous exposure to sodium fluoride. 

Lipid peroxidation is usually used as an indicator of oxidative stress in different tissues [58]. Malondialdehyde (MDA) represents the indicative end product of lipid peroxidation process in various tissues or biological fluids [59]. In the current study, a significant increase in the MDA level was observed in the gills, liver, kidney, and muscle tissues after exposure to fluoride. This increase in MDA levels represented an indicator for enhanced oxidative stress [60]. Induction of lipid peroxidation (LPO) and its related disturbance in the cell membranes’ integrity and inhibition of the membrane-bound enzymes was already reported previously [61].

In the current work, fish exposed to sodium fluoride (NaF) for eight weeks developed a significant reduction in the level of GSH and activity of SOD, CAT, and TAC in tissue homogenates of the liver, gills, kidney, and muscle. GSH is a main non-enzymatic endogenous antioxidant that prevents ROS and peroxide-induced cellular damage [62]. In addition, GSH works as a direct scavenger for free radicals and as a substrate for GPx and GST. Therefore, the reduction of GSH reported in this study may be attributed to its direct conjugation with the electrophiles generated due to sodium fluoride exposure. Similar reduction in the GSH level was previously reported in freshwater fish after exposure to other organophosphate methyl parathions [63]. 

SOD is an important antioxidant enzyme, it is considered as the first enzymatic defense against the superoxide anion. SOD is responsible for catalyzing the ROS binding with water to generate H_2_O_2_. Following that, the breakdown of H_2_O_2_ to water and oxygen is occurred via CAT to protect cells from the damaging effect of H_2_O_2_ and the hydroxyl radicals. These events may introduce an accepted explanation for the reduction in SOD and CAT level reported in the present study after exposure to fluoride. In addition, the observed reduction in SOD activity in fluoride-exposed fish may also be attributed to the direct competitive inhibition of enzyme activity by fluoride [64]. A similar finding was reported by many investigators [65,66]. In similar context, GPx is responsible for catalyzing the hydroperoxide reaction with reduced GSH and the resultant formation of glutathione disulfide. In the current work, exposure to sodium fluoride led to the reduction of the GPx activity in different tissues of sodium fluoride-exposed fish as compared to the control group. This finding may be attributed to the decreased activity of GSH which acts as a substrate for GPx. A similar finding was previously reported by Sharma et al. [67]. In addition, this reduction in GP_X_ activity could be attributed to the direct effects of metal ions on the active site of enzyme molecules. Waheed et al. [68] also indicated significant alterations in GPx activity together with SOD and CAT activities in the tissues of fish *Oreochromis niloticus* after Hg exposures. 

GSTs are a family of phase II detoxifying enzymes that are responsible for the conjugation of GSH to several electrophilic compounds [69]. In fish, GSTs play a major role in the antioxidant function and detoxification of various xenobiotics including fluoride. Several studies have been already reported that fluroacetamide reduced GST activity in various tissues due to oxidative stress [70,71]. This reduction of GST activity is directly related to increased levels of lipid peroxidation, where it plays a crucial role in eliminating MDA, the end-products of oxidative damage. Interestingly, the molecular expression of GST may be considered as a biomarker for xenobiotics exposure [72,73,74]. Many toxicologists expect that the highest level of GST expression may occur in the liver where metabolism mostly occurs [75]. In this study, the mRNA expression of GST in the liver of NaF-exposed fish showed a significant reduction compared to control fish. This reduction in GST may be related to the significant reduction of GSH, which is the substrate for GPx that provides a mechanism for GST involvement in the conjugation and elimination of fluoride [76]. In addition, it is well known that the GST enzyme catalyzes the reaction via the thiol (-SH) group of glutathione (GSH) leading to the neutralization of the xenobiotic and making it more water soluble [77]. However, supplementation of MO to NaF-exposed fish led to a significant elevation of GST mRNA expression in the liver. This elevation in GST activity might be attributed to the decline of free radical production by MO supplementation which, in turn, increases in the antioxidant system.

Co-administration of *Moringa oleifera* (MO) leaf extract led to a significant improvement of the antioxidative status of treated fish. The leaves of MO are rich in minerals (such as iron and calcium), vitamins (such as Vit. A, B and C), and proteins (such as methionine, cysteine, and essential sulfur amino acid) [78]. All parts of MO can act as good sources of natural antioxidants due to the presence of various types of antioxidant compounds, such as ascorbic acid, flavonoids, phenolics, and carotenoids [78]. In our study, the gas chromatography–mass spectrometry analysis of MO leaves revealed that they contain many antioxidant agents, such as eugenol (45.23%), caryophyllene (11.35%), hexadecanoic acid (7.23%), phenol (6.14%), octadecenoc acid (3.29%), heptadecyne (3.18%), cyclopropanoctonic (4.17%), heptatriacotanole (1.26%), and quercetin (0.89%). These natural antioxidant substances can illustrate the antioxidant property of MO against induced oxidative stress [79]. *Moringa oleifera* has been reported to exhibit efficient antioxidant and free-radical scavenging properties via lowering LPO levels in various tissues [32]. In this study, MO brought MDA levels to a normal level, and replenished the decreased level of GSH, SOD, and CAT, possibly due to its capability of scavenging the free radical generated after fluoride exposure. This replenishing of GSH may be responsible for the regaining of GPx and GST activity. The antioxidant capacity of MO has already been reported in some investigations; Onah et al., [32] concluded that supplementation of MO extracts is associated with a significant improvement in the levels of both liver and kidney GST, SOD, and CAT during lead acetate intoxication in fish [33]. Similarly, Uma et al. [34] and Fakurazi et al. [35] showed that MO leaves protected against acetaminophen-induced liver damage by decreasing liver enzymes and hepatic lipid peroxidation as well as increasing antioxidant enzyme levels. Similarly, MO improved MDA, SOD, GST, and CAT during lead acetate administration in fish [80]. The chemically-modified MO leaf powder was previously used by Reddy et al. [81] for optimization of Cd, Cu, and Ni biosorption. Removing of Cd from waste water was achieved using fresh leaves as biosorbents.

## 5. Conclusion and Limitation

On the basis of the present findings, it can be concluded that increased sodium fluoride content in water causes adverse oxidative stress on various fish tissues. The changes of tissue biomarkers as antioxidant enzymes were the physiological responses of *O. niloticus* to the stress of sodium fluoride exposure. *Moringa oleifera* 1% (*w*/*w*) ration is a promising plant-based antioxidant that may be grown to produce more natural products and materials against heavy metals toxicity and oxidation processes induced by NaF in various tissues of Nile tilapia. Estimation of growth data and the possibility of their modulation by oxidative stress induced by sodium fluoride and/or co-treatment with *Moringa oleifera*, as well as the measurement of water quality parameters, are important limitations of this study which must be considered in further studies.

## Figures and Tables

**Table 1:** Primer sequences used for RT-PCR.

Gene Name	Forward Primer	Reverse Primer
β-actin ^*^	CCTCACCCTCAAGTACCCCAT	TTGGCCTTTGGGTTGAGTG
*GST*	ATGATCTATGGCAACTATGAGACAGG	GAAGTACAAACAGATTGTATCCGC

* Housekeeping gene.

**Table 2:** GC-MS analysis of major phytoconstituents present in *Moringa oleifera* leaves ethanolic extract.

No.	Compound Name	RT (Minutes)	Relative Abundance (%)	Molecular Formula
1	Caryophyllene	15.88	11.35	C15H24
2	Eugenol	17.81	45.23	C10H12O2
3	Heptadecyne	20.25	3.18	C17H32O
4	Phenol	27.61	6.14	C12H14O3
5	Hexadecanoic acid	28.33	7.23	C38H68O8
6	Heptatriacotanole	28.42	1.26	C37H76O
7	Octadecenoic acid	29.56	3.29	C19H36O2
8	Quercetin	31.19	0.89	C18H16O7
9	Cyclopropanoctonic	31.24	2.17	C22H38O2

**Table 3:** Determination of sodium fluoride LC50 in *O. niloticus.*

Sodium Fluoride Dose (mg/L)	Overall Deaths within 96 h	A	B	AB
0 (control)	0	0	0	0
20	0	20	0	0
40	2	20	1.0	20
60	5	20	3.5	70
80	7	20	6.0	120
100	10	20	8.5	170
				∑ A × B = 390

A = differences between the two consecutive doses, B = arithmetic mean of the mortality caused by two consecutive doses. 96 h LC50 = LC100 – ∑ (A × B)/N = 100 – 390/10 = 61.0 ppm. or (mg/L). Lethal concentration 50 (LC50) of sodium fluoride in *O. niloticus* = 61 mg/L. A 1/10 dose of the LC50 of Sodium fluoride in *O. niloticus* to induce chronic toxicity was = 6.1 mg/L.

**Table 4:** Effect of Sodium fluoride (NaF) and/or *M. Oleifera (MO)* on malondialdehyde (MDA) (U/g tissue) in tissue homogenates of *O. niloticus* gills, liver, kidney, and muscle.

Groups	Period of Exposure				*p-*value
	2nd Week	4th Week	6th Week	8th Week	
**Gills**					
CTR	8.66 + 0.70 ^A a^	8.50 + 0.19 ^A b^	8.66 + 0.44 ^A c^	8.38 + 0.42 ^A b^	0.433
NaF	10.02 + 0.68 ^D a^	12.20+ 0.67 ^C a^	15.38 + 0.65 ^B a^	18.59 + 0.62 ^A a^	0.025
NaF + MO	9.95 + 0.22 ^C a^	11.32 + 0.30 ^C a^	13.07 + 0.33 ^B b^	17.34 + 0.79 ^A a^	0.038
MO	8.77 + 0.28 ^A a^	8.02 + 0.17 ^A,B b^	7.55 + 0.17 ^A,B c^	6.78 + 0.37 ^B b^	0.047
*p* value	0.091	0.023	0.001	0.028	
**Liver**					
CTR	11.68 + 0.73 ^A b^	11.67 + 0.54 ^A b^	10.78 + 0.42 ^A c^	11.54 + 0.67 ^A c^	0.970
NaF	14.00 + 0.34 ^D a^	17.88 + 0.34 ^C a^	26.65 + 0.80 ^B a^	38.30+0.67 ^A a^	0.001
NaF+MO	13.02 + 0.36 ^D a,b^	16.25 + 0.07 ^C a^	23.52 + 0.15 ^B b^	35.65 + 0.39 ^A b^	0.005
MO	11.44 + 0.05 ^A b^	10.23 + 0.27 ^A,B b^	9.60 + 0.15 ^B c^	7.77 + 0.41 ^C d^	0.014
*p* value	0.045	0.019	0.001	0.0001	
**Kidney**					
CTR	15.63 + 0.32 ^A b^	16.44 + 0.24 ^A b^	15.71 + 0.23 ^A c^	16.28 + 0.30 ^A c^	0.883
NaF	16.76 + 0.33 ^D a,b^	19.04 + 0.27 ^C a^	22.30 + 0.45 ^B a^	25.45 + 0.70 ^A a^	0.003
NaF+MO	17.07 + 0.26 ^C a^	17.09 + 0.43 ^C a^	20.70 + 0.50 ^B b^	23.79 + 0.39 ^A b^	0.011
MO	15.56 + 0.33 ^A b^	14.33 + 0.37 ^A,B c^	13.52 + 0.17 ^B d^	11.39 + 0.24 ^C d^	0.023
*p* value	0.041	0.001	0.001	0.001	
**Muscle**					
CTR	27.50 + 0.34 ^A a^	26.96 + 0.18 ^A b^	25.98 + 0.57 ^A c^	26.01 + 0.68 ^A c^	0.735
NaF	28.03 + 0.33 ^B a^	30.26 + 0.31 ^B a^	32.52 + 0.92 ^A a^	33.92 + 1.01 ^A a^	0.021
NaF+MO	27.58 + 0.17 ^C a^	28.29 + 0.58 ^B,C b^	29.75 + 0.38 ^A,B b^	30.90 + 0.66 ^A b^	0.031
MO	26.07 + 0.41 ^A a^	24.70 + 0.26 ^A,B c^	24.12 + 0.26 ^A,B c^	22.93 + 0.33 ^B d^	0.043
*p* value	0.212	0.028	0.006	0.001	

Different superscript small letters within the same column indicate significantly different mean values between different groups. Different superscript capital letters within the same row indicate significantly different mean values between different periods of exposure CTR, Control; NaF, sodium fluoride (6.1 mg/L.); NaF + MO, sodium fluoride (6.1 mg/L) + *Moringa oleifera* extract (1% in ration); MO, *M. oleifera* extract (1% in ration).

**Table 5:** Effect of Sodium fluoride (NaF) and/or *M. Oleifera (MO)* on glutathione reduced (GSH) (U/g tissue) in tissue homogenates of *O. niloticus* gills, liver, kidney, and muscle.

Groups	Period of Exposure				*p* value
	2nd Week	4th Week	6th Week	8th Week	
**Gills**					
CTR	5.25 + 0.041 ^Aa^	5.29 + 0.011 ^A a,b^	5.26 + 0.079 ^A, b^	5.26 + 0.055 ^A, b^	0.932
NaF	5.25 + 0.014 ^A a^	5.17 + 0.011 ^A,B b^	5.09 + 0.011 ^B c^	4.95 + 0.040 ^C d^	0.013
NaF + MO	5.24 + 0.032 ^A a^	5.23 + 1.017 ^A a,b^	5.25 + 0.038 ^A b^	5.20 + 0.069 ^A c^	0.843
MO	5.26 + 0.054 ^B a^	5.37 + 0.052 ^B a^	5.56 + 0.005 ^B a^	5.73 + 0.031 ^A a^	0.011
*p* value	0.748	0.043	0.017	0.001	
**Liver**					
CTR	6.69 + 0.027 ^A a^	6.67 + 0.032 ^A b^	6.65 + 0.034 ^A b^	6.72 + 0.024 ^A b^	0.736
NaF	6.37 + 0.035 ^A b^	6.19 + 0.011 ^B d^	5.86 + 0.043 ^C d^	5.46 + 0.046 ^D d^	0.037
NaF + MO	6.41 + 0.018 ^B b^	6.50 + 0.037 ^A c^	6.19 + 0.043 ^C c^	5.64 + 0.025 ^D c^	0.005
MO	6.79 + 0.034 ^D a^	6.97 + 0.080 ^C a^	7.39 + 0.023 ^B a^	7.86 + 0.044 ^A a^	0.001
*p* value	0.032	0.0001	0.008	0.0001	
**Kidney**					
CTR	2.59 + 0.017 ^A a^	2.49 + 0.028 ^A b^	2.50 + 0.034 ^A b^	2.55 + 0.035 ^A b^	0.683
NaF	2.53 + 0.026 ^A a^	2.39 + 0.008 ^B b^	2.34 + 0.051 ^B c^	2.18 + 0.050 ^C d^	0.037
NaF + MO	2.54 + 0.014 ^A a^	2.43 + 0.018 ^A,B b^	2.41 + 0.006 ^B b,c^	2.34 + 0.033 ^B c^	0.043
MO	2.63 + 0.010 ^C a^	2.70 + 0.014 ^B,C a^	2.82 + 0.048 ^A,B a^	2.92 + 0.060 ^A a^	0.048
*p* value	0.937	0.044	0.001	0.0007	
**Muscle**					
CTR	8.67 + 0.036 ^A a^	8.56 + 0.023 ^A b^	8.46 + 0.036 ^B b^	8.57 + 0.013 ^A b^	0.637
NaF	8.62 + 0.023 ^A a^	8.48 + 0.015 ^B b^	8.40+ 0.021 ^B b^	8.28 + 0.036 ^C d^	0.003
NaF + MO	8.62 + 0.055 ^A a^	8.53 + 0.017 ^A,B b^	8.43 + 0.017 ^B,C b^	8.39 + 0.011 ^C c^	0.047
MO	8.68 + 0.012 ^C a^	8.73 + 0.023 ^B,C a^	8.82 + 0.020 ^A,B a^	8.90 + 0.021 ^A a^	0.038
*p* value	0.078	0.031	0.020	0.001	

Different superscript small letters within the same column indicate significantly different mean values between different groups. Different superscript capital letters within the same row indicate significantly different mean values between different periods of exposure. CTR, control; NaF, sodium fluoride (6.1 mg/L.); NaF + MO, sodium fluoride (6.1 mg/L) + *Moringa oleifera* extract (1% in ration); MO, *M. oleifera* extract (1% in ration).

**Table 6:** Effect of sodium fluoride (NaF) and/or *M. Oleifera (MO)* on glutathione peroxidase (GPx) (U/g tissue) in tissue homogenates of *O. niloticus* gills, liver, kidney, and muscle.

Groups	Period of Exposure				*p* value
	2nd Week	4th Week	6th Week	8th Week	
**Gills**					
CTR	17.56 + 0.52 ^A a^	17.08 + 0.11 ^A a^	16.82 + 0.33 ^A a,b^	16.81 + 0.33 ^A a,b^	0.529
NaF	17.27 + 0.03 ^A a^	16.62 + 0.12 ^A.B a^	16.40 + 0.06 ^A.B b^	16.27 + 0.02 ^B b^	0.037
NaF+MO	17.43 + 0.06 ^A a^	16.74 + 0.11 ^A,B a^	16.52 + 0.08 ^A,B a,b^	16.41 + 0.05 ^B b^	0.022
MO	17.62 + 0.53 ^A a^	17.68 + 0.51 ^A a^	17.73 + 0.72 ^A a^	18.00 + 0.22 ^A a^	0.693
*p* value	0.079	0.320	0.033	0.047	
**Liver**					
CTR	27.77 + 0.37 ^A a^	27.52 + 0.29 ^A a^	27.78 + 0.34 ^A a^	26.84 + 0.22 ^A b^	0.683
NaF	26.27 + 0.05 ^A b^	24.78 + 0.25 ^B b^	22.45 + 0.34 ^C b^	18.913 + 0.74 ^D c^	0.002
NaF+MO	26.43 + 0.05 ^A b^	24.83 + 0.65 ^B b^	22.85 + 0.22 ^C b^	19.49 + 0.19 ^D c^	0.001
MO	27.87 + 0.39 ^B a^	28.62 + 0.04 ^A,B a^	28.89 + 0.05 ^A,B a^	29.21 + 0.02 ^A a^	0.036
*p* value	0.019	0.022	0.014	0.009	
**Kidney**					
CTR	22.97 + 0.37 ^A a^	22.06 + 0.16 ^B b^	22.38 + 0.47 ^A, b^	22.89 + 0.25 ^A b^	0.660
NaF	22.28 + 0.12 ^A a^	21.71 + 0.20 ^A,B b^	21.65 + 0.05 ^A,B b^	21.28 + 0.07 ^B b^	0.048
NaF+MO	22.36 + 0.11 ^A a^	21.81 + 0.23 ^A b^	21.84 + 0.04 ^A b^	21.66 + 0.06 ^A b^	0.584
MO	23.04 + 0.36 ^A a^	23.20 + 0.29 ^A a^	23.52 + 0.32 ^A a^	23.59 + 0.33 ^A a^	0.408
*p* value	0.849	0.039	0.022	0.019	
**Muscle**					
CTR	15.39 + 0.09 ^B a^	15.95 + 0.37 ^A,B a^	16.26 + 0.30 ^A a^	15.82 + 0.45 ^A,B a^	0.052
NaF	15.29 + 0.11 ^A a^	15.19 + 0.01 ^A b^	15.12 + 0.01 ^A b^	14.87 + 0.15 ^A b^	0.096
NaF+MO	15.33 + 0.10 ^A a^	15.45 + 0.09 ^A a,b^	15.43 + 0.08 ^A b^	15.00 + 0.11 ^A b^	0.192
MO	15.46 + 0.08 ^A a^	15.53 + 0.07 ^A a,b^	15.69 + 0.01 ^A a,b^	15.88 + 0.01 ^A a^	0.210
*p* value	0.091	0.051	0.044	0.039	

Different superscript small letters within the same column indicate significantly different mean values between different groups. Different superscript capital letters within the same row indicate significantly different mean values between different periods of exposure. CTR, control; NaF, sodium fluoride (6.1 mg/L.); NaF + MO, sodium fluoride (6.1 mg/L) + *Moringa oleifera* extract (1% in ration); MO, *M. oleifera* extract (1% in ration).

**Table 7:** Effect of Sodium fluoride (NaF) and/or *M. Oleifera (MO)* on catalase (CAT) (U/g tissue) in tissue homogenates of *O. niloticus* gills, liver, kidney, and muscle.

Groups	Period of Exposure				*p* value
	2nd Week	4th Week	6th Week	8th Week	
**Gills**					
CTR	7.84 + 0.04 ^A a^	7.76 + 0.04 ^A b^	7.65 + 0.03 ^A b^	7.85 + 0.03 ^A b^	0.683
NaF	7.77 + 0.14 ^A a^	7.59 + 0.02 ^A,B b^	7.46 + 0.05 ^B,C b^	7.27 + 0.04 ^C d^	0.019
NaF + MO	7.79 + 0.06 ^A a^	7.67 + 0.02 ^A b^	7.54 + 0.04 ^A b^	7.54 + 0.02 ^A c^	0.747
MO	7.97 + 0.06 ^B a^	8.07 + 0.06 ^B a^	8.20 + 0.05 ^A,B a^	8.32 + 0.08 ^A a^	0.028
*p* value	0.089	0.031	0.025	0.001	
**Liver**					
CTR	18.78 + 0.3 ^B a^	19.01 + 0.3 ^A,B a^	18.97 + 0.4 ^B b^	20.21 + 0.3 ^A a^	0.048
NaF	18.54 + 0.3 ^A a^	16.70 + 0.2 ^B b^	14.99 + 0.2 ^C c^	13.58 + 0.2 ^D b^	0.001
NaF + MO	18.69 + 0.3 ^A a^	17.24 + 0.3 ^B b^	14.66 + 0.3 ^C c^	14.28 + 0.04 ^C b^	0.007
MO	18.92 + 0.3 ^C a^	19.37 + 0.1 ^B,C a^	20.23 + 0.5 ^A,B a^	20.71 + 0.5 ^A a^	0.021
*p* value	0.397	0.020	0.016	0.009	
**Kidney**					
CTR	36.34 + 0.6 ^B a^	36.58 + 0.7 ^B a^	37.55 + 0.6 ^A a^	38.81 + 0.4 ^A a^	0.049
NaF	36.29 + 0.6 ^A a^	36.17 + 0.03 ^A a^	35.67 + 0.1 ^A b^	35.63 + 0.2 ^A b^	0.102
NaF + MO	36.32 + 0.6 ^A a^	36.32 + 0.05 ^A a^	35.90 + 0.1 ^A a,b^	35.81 + 0.08 ^A b^	0.937
MO	36.39 + 0.5 ^A a^	36.57 + 0.5 ^A a^	36.62 + 0.7 ^A a,b^	36.54 + 0.3 ^A b^	0.776
*p* value	0.556	0.325	0.046	0.027	
**Muscle**					
CTR	16.96 + 0.8 ^B a^	17.54 + 0.08 ^B a^	17.46 + 46 ^B a^	20.12 + 0.9 ^A a^	0.010
NaF	16.91 + 0.8 ^A a^	16.64 + 0.3 ^A a^	16.35 + 0.4 ^A a^	15.95 + 42 ^A b^	0.087
NaF + MO	16.97 + 0.8 ^A a^	16.87 + 0.2 ^A a^	16.67 + 0.4 ^A a^	16.06 + 0.3 ^A b^	0.265
MO	17.03 + 0.8 ^A a^	17.24 + 0.8 ^A a^	17.21 + 0.6 ^A a^	17.29 + 0.8 ^A b^	0.563
*p* value	0.658	0.547	0.172	0.028	

Different superscript small letters within the same column indicate significantly different mean values between different groups. Different superscript capital letters within the same row indicate significantly different mean values between different periods of exposure. CTR, control; NaF, sodium fluoride (6.1 mg/L.); NaF + MO, sodium fluoride (6.1 mg/L) + *Moringa oleifera* extract (1% in ration); MO, *M. oleifera* extract (1% in ration).

**Table 8:** Effect of sodium fluoride (NaF) and/or *M. Oleifera (MO)* on super oxide dismutase (SOD) (U/g tissue) in tissue homogenates of *O. niloticus* gills, liver, kidney, and muscle.

Groups	Period of Exposure				*p* value
	2nd Week	4th Week	6th Week	8th Week	
**Gills**					
CTR	284.38 + 5.85 ^B b^	304.67 + 4.14 ^A a^	282.34 + 1.57 ^B b^	277.01 + 0.91 ^B b^	0.002
NaF	276.19 + 3.53 ^A b^	269.62 + 2.65 ^A,B b^	264.49 + 2.15 ^B,C c^	258.99 + 0.91 ^C c^	0.023
NaF + MO	280.25 + 4.07 ^A b^	276.86 + 2.59 ^A b^	273.80 + 1.10 ^A,B c^	265.37 + 1.54 ^B c^	0.037
MO	300.81 + 5.47 ^C a^	311.52 + 1.29 ^B a^	317.08 + 2.72 ^A,B a^	323.94 + 1.73 ^A a^	0.210
*p* value	0.036	0.021	0.013	0.001	
**Liver**					
CTR	390.00 + 2.54 ^A a,b^	387.58 + 2.84 ^A b^	380.34 + 1.86 ^A,B b^	372.10 + 1.52 ^B b^	0.048
NaF	373.70 + 2.49 ^A c^	348.85 + 2.86 ^B c^	323.18 + 2.27 ^C c^	304.41 + 5.35 ^D c^	0.031
NaF + MO	381.56 + 4.37 ^A b,c^	351.67 + 4.09 ^B c^	330.39 + 1.47 ^C c^	323.07 + 2.53 ^C c^	0.028
MO	396.36 + 2.96 ^D a^	411.38 + 0.45 ^C a^	423.79 + 1.96 ^B a^	437.34 + 3.13 ^A a^	0.011
*p* value	0.022	0.001	0.012	0.0009	
**Kidney**					
CTR	715.59 + 1.40 ^A a^	711.53 + 4.93 ^A b^	714.92 + 6.52 ^A b^	707.14 + 2.26 ^A b^	0.656
NaF	710.18 + 0.55 ^A a^	693.25 + 2.25 ^B c^	681.60 + 1.23 ^C c^	675.67 + 1.43 ^C c^	0.026
NaF + MO	711.48 + 1.47 ^A a^	704.74 + 4.17 ^A b^	686.00 + 0.92 ^B c^	686.25 + 1.27 ^B c^	0.033
MO	719.35 + 0.49 ^B a^	722.18 + 0.77 ^B a^	725.66 + 1.29 ^A,B a^	732.73 + 0.88 ^A a^	0.048
*p* value	0.066	0.024	0.013	0.001	
**Muscle**					
CTR	176.66 + 2.52 ^A,B a,b^	178.53 + 1.99 ^A a^	170.60 + 1.27 ^B,C b^	168.76 + 3.44 ^C b^	0.049
NaF	170.75 + 2.05 ^A b^	170.22 + 0.85 ^A b^	159.45 + 1.08 ^B c^	155.55 + 1.82 ^B c^	0.031
NaF + MO	171.01 + 0.80 ^A b^	167.35 + 0.33 ^A,B b^	164.29 + 0.64 ^A,B b,c^	162.58 + 0.96 ^B b,c^	0.044
MO	182.36 + 1.29 ^B a^	182.99 + 2.79 ^B a^	187.99 + 2.48 ^A,B a^	190.54 + 2.43 ^A a^	0.026
*p* value	0.031	0.029	0.019	0.015	

Different superscript small letters within the same column indicate significantly different mean values between different groups. Different superscript capital letters within the same row indicate significantly different mean values between different periods of exposure. CTR, control; NaF, sodium fluoride (6.1 mg/L.); NaF + MO, sodium fluoride (6.1 mg/L) + *Moringa oleifera* extract (1% in ration); MO, *M. oleifera* extract (1% in ration).

**Table 9:** Effect of sodium fluoride (NaF) and/or *M. Oleifera (MO)* on total antioxidant capacity (TAC) (U/g tissue) in tissue homogenates of *O. niloticus* gills, liver, kidney, and muscle.

Groups	Period of Exposure				*p* value
	2nd Week	4th Week	6th Week	8th Week	
**Gills**					
CTR	1.45 + 0.005 ^B a,b^	1.42 + 0.005 ^C b^	1.47 + 0.005 ^B b^	1.51 + 0.009 ^A b^	0.037
NaF	1.42 + 0.008 ^A a,b^	1.39 + 0.008 ^B b^	1.32 + 0.017 ^C c^	1.20 + 0.025 ^D d^	0.021
NaF + MO	1.39 + 0.008 ^A b^	1.40 + 0.001 ^A b^	1.37 + 0.008 ^A c^	1.38 + 0.014 ^A c^	0.072
MO	1.46 + 0.043 ^D a^	1.51 + 0.023 ^C a^	1.58 + 0.024 ^B a^	1.67 + 0.020 ^A a^	0.001
*p* value	0.023	0.019	0.008	0.009	
**Liver**					
CTR	4.73 + 0.011 ^A,B a^	4.71 + 0.048 ^B a^	4.73 + 0.024 ^A,B b^	4.82 + 0.023 ^A b^	0.046
NaF	4.64 + 0.006 ^A a^	4.48 + 0.023 ^B b^	4.26 + 0.025 ^C d^	4.00 + 0.071 ^D d^	0.001
NaF + MO	4.67 + 0.008 ^A a^	4.55 + 0.041 ^B b^	4.57 + 0.018 ^A,B c^	4.62 + 0.020 ^A,B c^	0.032
MO	4.75 + 0.010 ^C a^	4.80 + 0.005 ^C a^	4.92 + 0.020 ^B a^	5.28 + 0.015 ^A a^	0.026
*p* value	0.089	0.026	0.024	0.011	
**Kidney**					
CTR	3.88 + 0.017 ^A a^	3.82 + 0.027 ^A,B b^	3.82 + 0.015 ^B b^	3.88 + 0.014 ^A b^	0.041
NaF	3.85 + 0.009 ^A a^	3.78 + 0.005 ^B b^	3.73 + 0.005 ^B c^	3.66 + 0.006 ^C d^	0.010
NaF + MO	3.86 + 0.020 ^A a^	3.83 + 0.015 ^A,B b^	3.77 + 0.009 ^B b,c^	3.79 + 0.018 ^B c^	0.038
MO	3.88 + 0.012 ^C a^	3.93 + 0.003 ^C a^	4.03 + 0.026 ^B a^	4.16 + 0.025 ^A a^	0.018
*p* value	0.073	0.044	0.028	0.038	
**Muscle**					
CTR	6.81 + 0.011 ^B,C a^	6.78 + 0.012 ^C b^	6.85 + 0.015 ^A,B b^	6.87 + 0.023 ^A b^	0.036
NaF	6.77 + 0.005 ^A a,b^	6.72 + 0.003 ^A c^	6.65 + 0.006 ^B d^	6.59 + 0.005 ^C d^	0.033
NaF + MO	6.75 + 0.005 ^A a,b^	6.76 + 0.010 ^A b,c^	6.79 + 0.005 ^A c^	6.78 + 0.020 ^A c^	0.078
MO	6.80 + 0.012 ^C a^	6.84 + 0.008 ^C a^	6.92 + 0.020 ^B a^	6.98 + 0.029 ^A a^	0.011
*p* value	0.039	0.012	0.017	0.011	

Different superscript small letters within the same column indicate significantly different mean values between different groups. Different superscript capital letters within the same row indicate significantly different mean values between different periods of exposure. CTR, control; NaF, sodium fluoride (6.1 mg/L.); NaF + MO, sodium fluoride (6.1 mg/L) + *Moringa oleifera* extract (1% in ration); MO, *M. oleifera* extract (1% in ration).

## Abbreviations

NaF	Sodium fluoride
*O. niloticus*	*Oreochromis niloticus*
MO	*Moringa oleifera*
LC50	Lethal concentration 50
MDA	Malondialdehyde
CAT	Catalase
SOD	Super oxide dismutase
GPx	Glutathione peroxidase
GST	Glutathione S transferase
GSH	Glutathione reduced
TAC	Total antioxidant capacity
